# The Role of the Component Metals in the Toxicity of Military-Grade Tungsten Alloy

**DOI:** 10.3390/toxics3040499

**Published:** 2015-12-08

**Authors:** Christy A. Emond, Vernieda B. Vergara, Eric D. Lombardini, Steven R. Mog, John F. Kalinich

**Affiliations:** 1 Internal Contamination and Metal Toxicity Program, Armed Forces Radiobiology Research Institute, Uniformed Services University, 8901 Wisconsin Ave, Bethesda, MD 20889-5603, USA; E-Mails: christy.emond@va.gov (C.A.E.); vernieda@yahoo.com (V.B.V.); 2 Division of Comparative Pathology, Veterinary Sciences Department, Armed Forces Radiobiology Research Institute, Uniformed Services University, 8901 Wisconsin Ave, Bethesda, MD 20889-5603, USA; E-Mails: eric.d.lombardini.mil@us.army.mil (E.D.L.); steven.mog@fda.hhs.gov (S.R.M.)

**Keywords:** tungsten alloy, B6C3F_1_ mice, embedded metal fragments, health effects

## Abstract

Tungsten-based composites have been recommended as a suitable replacement for depleted uranium. Unfortunately, one of these mixtures composed of tungsten (W), nickel (Ni) and cobalt (Co) induced rhabdomyosarcomas when implanted into the leg muscle of laboratory rats and mice to simulate a shrapnel wound. The question arose as to whether the neoplastic effect of the mixture could be solely attributed to one or more of the metal components. To investigate this possibility, pellets with one or two of the component metals replaced with an identical amount of the biologically-inert metal tantalum (Ta) were manufactured and implanted into the quadriceps of B6C3F_1_ mice. The mice were followed for two years to assess potential adverse health effects. Implantation with WTa, CoTa or WNiTa resulted in decreased survival, but not to the level reported for WNiCo. Sarcomas in the implanted muscle were found in 20% of the CoTa-implanted mice and 5% of the WTa- and WCoTa-implanted rats and mice, far below the 80% reported for WNiCo-implanted mice. The data obtained from this study suggested that no single metal is solely responsible for the neoplastic effects of WNiCo and that a synergistic effect of the three metals in tumor development was likely.

## 1. Introduction

Advances in military weapons’ design has led to an increase in the complexity of metal mixtures on today’s battlefields. Gone are the days when lead and iron were the only concerns with respect to embedded fragment wounds. The ability of armored vehicles to withstand penetration by projectiles led to the development of more effective armor-penetrating munitions. One material in this quest proved to be superior, depleted uranium. Depleted uranium (DU) is the by-product of the process of producing enriched uranium from natural uranium [1]. Natural uranium primarily consists of three isotopes in the following percentages: ^238^U (99.274%), ^235^U (0.720%) and ^234^U (0.006%). The process to obtain reactor- and weapon-grade uranium results in a product with an increased percentage of ^234^U and ^235^U, but also a by-product that is “depleted” with respect to these isotopes. DU consists of 99.745% ^238^U, 0.250% ^235^U and 0.005% ^234^U. Thus, it has a lower specific activity than natural uranium, yet still retains the same chemical properties. These properties make DU an excellent material for armor-penetrating munitions.

DU munitions saw their first widespread combat use during the First Persian Gulf War. Despite their acknowledged success, concern was raised over the long-term health and environmental effects of their use. As a result, materials that could replace DU in armor penetrators were sought, and several tungsten-based compositions were shown to be promising. Because of its high density and melting point, tungsten was already used in a wide variety of other commercial and military applications [2]. Tungsten has been used in light bulb filaments, thermocouples, counterweights and radiation shields. It is also found extensively, as tungsten carbide, in drill bits and cutting blades. Because of the concern over lead poisoning in waterfowl resulting from ingestion of spent ammunition, the United States Fish and Wildlife Service banned the use of lead-based ammunition for the hunting of waterfowl in the early 1990s [3]. The recommended alternatives contained varying amounts of tungsten (40%–60%) in combination with other metals, such as nickel, tin, iron, copper and bismuth. Subsequent toxicity testing has shown no adverse health effects of these materials when administered through a variety of exposure scenarios [4,5,6,7,8,9].

Military applications also include the use of tungsten-based composites, primarily tungsten/tin and tungsten/Nylon, as proposed replacements for lead in small-caliber ammunition [10]. As noted earlier, concern over the health and environmental impact of the use of depleted uranium has led many countries to replace depleted uranium with various tungsten alloys in their arsenals of armor-penetrating munitions. In many of these formulations, tungsten is combined with two or more of the following transition metals: nickel, cobalt, iron and copper. Unfortunately, one of these, an alloy of 91.1% tungsten (W), 6% nickel (Ni) and 2.9% cobalt (Co), was shown to induce rhabdomyosarcomas when surgically implanted in the leg muscles of laboratory rats and mice to simulate a shrapnel wound [11,12,13]. Conversely, implantation of an alloy of 91% tungsten, 7% nickel and 2% iron (Fe) did not result in any adverse health effects [12,13]. This raises the question of whether one or more of the component metals can be identified as the cause of rhabdomyosarcoma formation. This study attempts to address this by replacing each of the metals in the alloy with tantalum, a biologically-inert metal that has been previously used in human prostheses [14,15,16]. The manufactured pellets were then surgically implanted into the quadriceps muscles of the B6C3F_1_ laboratory mouse, and the mice were followed for two years for signs of adverse health effects.

## 2. Experimental Section

### 2.1. Animals

Male B6C3F_1_ mice (3–4 weeks old) were supplied by Harlan (Dublin, VA, USA). All animals were maintained in an Association for Assessment and Accreditation of Laboratory Animal Care International (AAALAC-I)-accredited facility in accordance with the Guide for the Care and Use of Laboratory Animals [17]. The Armed Forces Radiobiology Research Institute’s (AFRRI) Animal Care and Use Committee (IACUC) approved all animal-related procedures prior to initiation. Mice were group-housed (5 mice/group) in plastic microisolator cages with hardwood chips for bedding and fed standard rodent chow, with water provided *ad libitum*. Animals were on a 12-h light/dark cycle. Health status was monitored daily, and animals were weighed weekly.

### 2.2. Metal Pellets

The pellets used in this project were cylindrical in shape (1 mm in diameter by 2 mm in length). Tantalum (99.95% Ta) pellets were obtained from Alfa Aesar (Ward Hill, MA, USA). Tantalum was selected as the implantation control metal, because it is considered biologically inert [14,15,16]. The remaining pellets were manufactured at Aerojet Ordnance Tennessee (Jonesborough, TN, USA). When selected metals were omitted from a pellet’s composition, tantalum was substituted to keep pellet dimensions the same and weights similar. The metal composition of the manufactured pellets is shown in Table 1. Before implantation, pellets were cleaned and chemically sterilized as previously described [13].

### 2.3. Pellet-Implantation Procedures

Anesthesia was induced via isoflurane inhalation. The implantation sites were clipped and washed with betadine. A small incision was made through the skin using a scalpel to expose the quadriceps. Two pellets were implanted into each quadriceps. Incision sites were closed with tissue adhesive (VetBond, 3M Corp., St. Paul, MN, USA). The mice were closely monitored after pellet implantation until they were ambulatory, and an analgesic (buprenorphine, 0.1 mg/kg, sc, Reckitt and Benckiser, Hull, UK) was administered as needed. The pellet implantation sites were examined daily for signs of inflammation and infection for two weeks after surgery, then weekly thereafter until completion of the study.

### 2.4. Experimental Groups

The pellet implantation groups included a surgical sham, tantalum and six metal combinations. Five experimental time points were run: 1, 3, 6, 12 and 24 months post-implantation. Upon reaching their experimental endpoint or when necessitated by IACUC-approved health guidelines, mice were euthanized by isoflurane administration followed by exsanguination and confirmatory pneumothorax. A necropsy examination was conducted and a variety of tissues isolated for analysis. The wet weights of liver, spleen, kidney and testes were determined and normalized to body weight. A blood sample for hematological assessments and serum isolation was collected by cardiac puncture. In addition, a urine sample for metal analysis was removed from the bladder. Urine and serum samples were stored at −80 °C until assayed.

### 2.5. Hematology

A ScilVet Hematology Analyzer (Scil Animal Care Company, Gurnee, IL, USA) was used to obtain a variety of hematological parameters, including white and red blood cell counts, hemoglobin, hematocrit, as well as platelet, lymphocyte, monocytes and granulocyte counts from the collected blood samples.

### 2.6. Pathology

Tissues for histopathology were fixed in buffered zinc formalin, processed and embedded in paraffin, sectioned at 5–6 µm and stained with hematoxylin and eosin before microscopic examination by board-certified veterinary pathologists.

### 2.7. Sample Preparation for Metal Analysis

The collected samples needed various amounts of processing before metal determination. Urine samples required only dilution prior to analysis. Serum samples were wet-ashed with 5 mL of 70% nitric acid (Optima Ultrapure Grade, Fisher Scientific, Pittsburgh, PA, USA) and 200 µL of 30% hydrogen peroxide (Semiconductor Grade, Sigma-Aldrich Chemical Co., St. Louis, MO, USA), then heated to just below boiling until completely evaporated. The samples were then dry-ashed for 12 h at 600 °C in a muffle furnace (Fisher Scientific) prior to another cycle of wet-ashing with nitric acid and hydrogen peroxide. After the second run of wet-ashing, the resulting white residue was dissolved in 2% nitric acid and analyzed. Tissue samples required more extensive processing, which included first drying the tissue in a muffle furnace at 100 °C for 24 h. The temperature of the furnace was then ramped to 350 °C, at 5 °C/min, and the samples held there for 24 h. The furnace temperature was then ramped to 600 °C, at 5 °C/min, and the samples held there for 48 h. After cooling, the samples were wet- and dry-ashed as described above.

### 2.8. Metal Analysis

Inductively-coupled plasma-mass spectrometry was used for the determination of metal content. An X Series 2 Inductively-Coupled Plasma-Mass Spectrometer (ThermoElectron North America, LLC., Madison, WI, USA) equipped with a Cetac ASX520 Autosampler (Cetac Technologies, Omaha, NE, USA) was utilized. The plasma gas was high-purity (99.997%) liquid argon. The appropriate metal standards obtained from SPEX CertiPrep (Metuchen, NJ, USA) in 2% HNO_3_ were used for instrument calibration. Metal concentration levels were obtained by reference to the slope of the calibration curve (counts per second/ng per liter), as well as an internal standard [13]. Urine data were normalized to creatinine levels with creatinine content determined by a modified Jaffe reaction [18,19] and a commercially available kit (Oxford Biomedical Research, Oxford, MI, USA).

### 2.9. Statistical Analysis

The Kaplan–Meier procedure [20] was used to graph survival data with median survival times analyzed by the Wilcoxon–Mann–Whitney *U*-test to determine statistical significance. Other data were analyzed by a one-way ANOVA to assess the effect of metal implantation. If a significant effect were observed, Dunnett’s tests were conducted to determine which groups were statistically different from the non-implanted control, with *p* < 0.05 considered statistically significant.

## 3. Results and Discussion

### 3.1. Pellet Formulations

In order to determine if the carcinogenic effect observed with embedded WNiCo pellets could be attributed to a particular metal, pellets were reformulated to remove one or two of the components metals and to replace them with an equal percentage of tantalum, a biologically-inert metal. Aerojet Ordnance manufactured the WNiCo pellets used in an earlier study [13] and also produced the following pellets for this investigation: WTa, NiTa, CoTa, WNiTa, WCoTa and NiCoTa. The percentages of the various components are shown in Table 1.

**Table 1:** Pellet composition.

Group	Metal (%)			
Pellet	W	Ni	Co	Ta
Ta	-	-	-	100
WTa	91.1	-	-	8.9
NiTa	-	6.0	-	94.0
CoTa	-	-	2.9	97.1
WNiTa	91.1	6.0	-	2.9
WCoTa	91.1	-	2.9	6.0
NiCoTa	-	6.0	2.9	91.1

The cylindrical pellets (1 mm × 2 mm) were chemically sterilized prior to implantation surgery, as previously described [13].

### 3.2. Pellet Implantation

**Figure 1:**
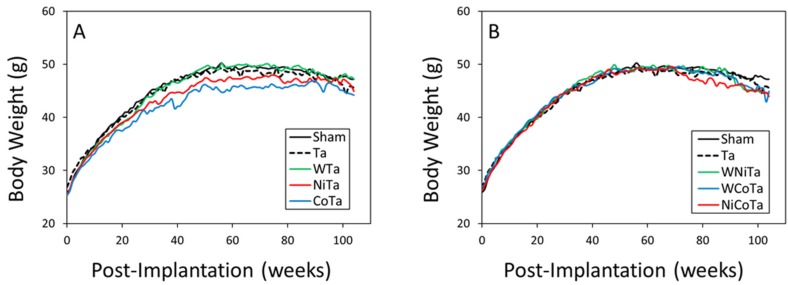
Body weight gain. (**A**) One-metal groups; (**B**) two-metal groups.

Pellets were surgically implanted into the quadriceps muscles of male B6C3F_1_ mice with two pellets implanted per leg. This technique was developed at our institute and has been used extensively over the years to investigate the health effects of a variety of embedded metal fragments [1,11,13,20,21]. The quadriceps was selected as the site of implantation instead of the gastrocnemius, as had been used for the rat, because of the larger muscle mass of the quadriceps in the mouse. The mice recovered quickly from the surgery, and because of the small size of the pellets, no effects on mobility and normal behavior were observed. The surgical sites were examined daily for the first two weeks post-surgery and then weekly thereafter for signs of infection, inflammation and local metal toxicity. Furthermore, starting at two weeks post-implantation and continuing for the life of the animal, the pellet implantation sites were palpated to assess any abnormal tissue growth. Individual body weights were also recorded weekly. Body weight gain is an excellent indicator of overall health in rodents. As shown in Figure 1, all experimental groups initially gained weight at a similar rate. However, starting at 15 weeks post-implantation, weight gain in the CoTa group slowed significantly. In addition, weight gain also slowed in the NiTa and NiCoTa groups starting at 31 and 75 weeks, respectively.

### 3.3. Survival Rates and Necropsy Findings

**Figure 2:**
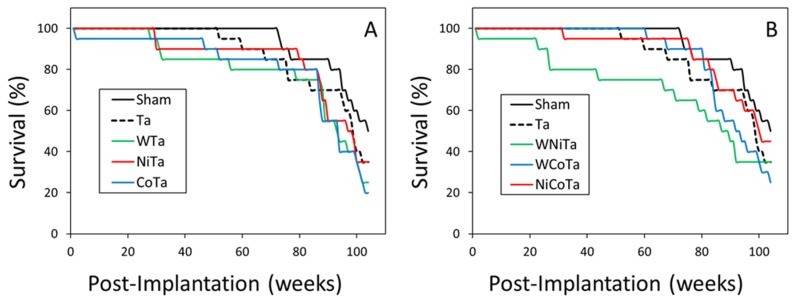
Kaplan–Meier survival plots for the one-metal (**A**) and two-metal (**B**) groups.

Figure 2 shows the survival of the one-metal and two-metal experimental groups throughout the project. All of the experimental groups showed a shorter median lifespan than the non-implanted control group. However, this decrease was only statistically significant for three groups. As seen in Table 2, the WTa, CoTa and WNiTa groups all had significantly shorter median lifespans than the control. As a point of comparison, B6C3F_1_ mice implanted with four pellets of WNiCo had a mean survival time of 72 weeks [13].

**Table 2:** Median survival times of metal-implanted mice.

Group	Median Survival (Weeks)
Sham	104
Ta	99
WTa	93 *
NiTa	97
CoTa	93 *
WNiTa	87 *
WCoTa	92
NiCoTa	100

All mice were implanted with 4 pellets. Median survival calculated from *n* = 20 for each experimental group. Values statistically different from sham control group at *p* < 0.5, as determined by the Wilcoxon–Mann–Whitney *U*-test, are denoted by *.

Upon examination at necropsy, a number of neoplasms were noted in various tissues. These are summarized in Table 3. Except for skeletal muscle, the presence, both in location, as well as type, of the tumors was not surprising and correlated to findings on spontaneous tumors in the B6C3F_1_ mouse from a wide range of studies [22,23]. Hepatocellular adenomas and carcinomas comprised the neoplasms found in liver, while those in lung were identified as bronchiolo-alveolar adenomas and carcinomas. Lymphomas and clear cell carcinomas were found in kidney, and sarcomas and lymphomas were identified in spleen. The sole testicular neoplasm was an interstitial cell tumor. The percentages and types of neoplasms from the mice in these study were no different than those reported from other investigations [22,23].

**Table 3:** Tumor locations in 24-month experimental groups.

Tissue						
Group	Liver	Lung	Spleen	Kidney	Testes	Skeletal Muscle
Sham	8	4	1	-	-	-
Ta	8	2	1	2	-	-
WTa	8	6	-	-	-	1
NiTa	8	5	1	-	-	-
CoTa	7	2	2	-	-	4
WNiTa	5	1	1	1	1	-
WCoTa	7	-	2	-	-	1
NiCoTa	6	1	3	1	-	-

Results are the total number of tumors found in tissues from animals in the 24-month experimental groups (*n* = 20 per group).

**Figure 3:**
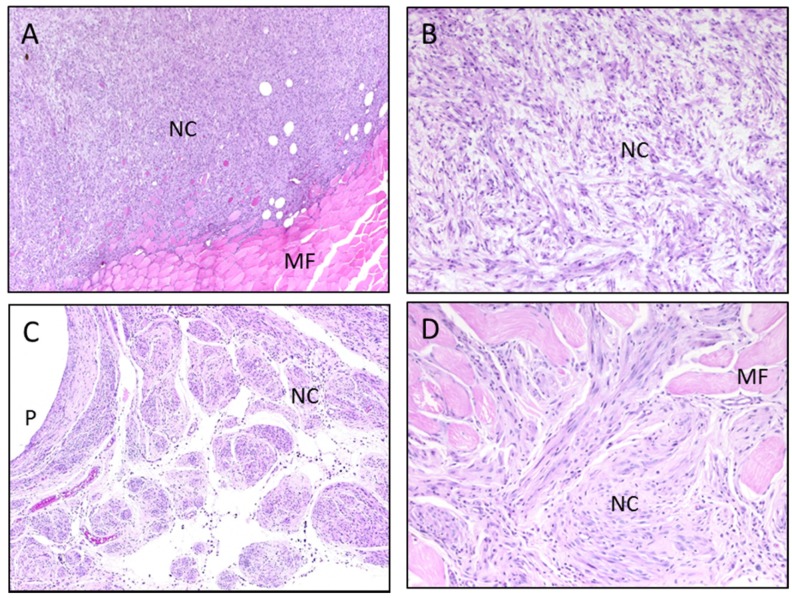
Histopathologic examination of leg tumors in 24-month metal-implanted mice. (**A**,**B**) H&E-stained section of tumor surrounding WTa pellet; (**C**,**D**) H&E-stained section of tumor surrounding CoTa pellet; (**A**,**C**) magnification ×40; (**B**,**D**) magnification ×100. NC, neoplastic cells; MF, normal muscle fiber; P, pellet hole; H&E, hematoxylin and eosin; W, tungsten; Co, cobalt; Ta, tantalum.

The skeletal muscle neoplasms found in the WTa-, CoTa- and WCoTa-implanted groups were identified as malignant sarcomas, two with morphological features of malignant schwannoma (Figure 3). Spontaneous skeletal muscle tumors are exceedingly rare in the B6C3F_1_ mouse (<0.1%) [23]; thus, the 5%–20% incidence found in the WTa-, CoTa- and WCoTa-implanted groups was significant. However, it pales in comparison with the 80% tumor incidence found in WNiCo-implanted mice [13]. In addition, the WNiCo-induced tumors were palpable, on average, at 48 weeks post-implantation. While the single WCoTa-induced tumor was palpable starting at 24 weeks post-implantation, neither the WTa- nor CoTa-induced tumors reached a mass that could be distinguished by manual palpation.

Organ-body weight ratios provide an indication of organ-specific toxicity. No long-term differences in organ-body weight ratios were observed for liver, spleen, kidney or testes across any of the experimental implantation groups. In addition, no long-lasting hematological changes were found across the experimental groups with respect to white and red blood cell counts, hemoglobin, hematocrit and platelet, lymphocyte, monocytes and granulocyte counts. Organ-body weight ratio and hematological results for all experimental groups can be found in the Supplementary Data Tables S1–S18.

### 3.4. Tissue Metal Analysis

Upon euthanasia, a variety of tissues, including brain, liver, spleen, kidney, femur and testes, was collected for metal analysis. In addition, samples of serum and urine were also obtained for metal analysis. After preparation, samples were analyzed for tungsten, nickel, cobalt and tantalum using inductively-coupled plasma-mass spectrometry (ICP-MS). Instrument operating conditions and parameters are given in Table 4.

**Table 4:** ICP-MS operating conditions and parameters.

*Instrument Parameters*	
Nebulizer type	Concentric
Spray chamber	Conical, with impact bead
Sampler cone	Platinum, 1-mm orifice diameter
Skimmer cone	Platinum, 0.7-mm orifice diameter
Sample uptake rate	1.0 mL/min
Sample read delay	60 s
*Plasma Conditions*	
RF power	1400 W
Plasma argon gas flow	13.0 L/min
Auxiliary argon gas flow	0.80 L/min
Nebulizer gas flow	0.94 L/min
*Mass Spectrometer Settings*	
Scanning mode	Peak jump
Sweeps	100
Dwell time	600 µs
Channels/mass	1
Acquisition time	18 s
Number of readings/replicate	5
Number of replicates	2

**Figure 4:**
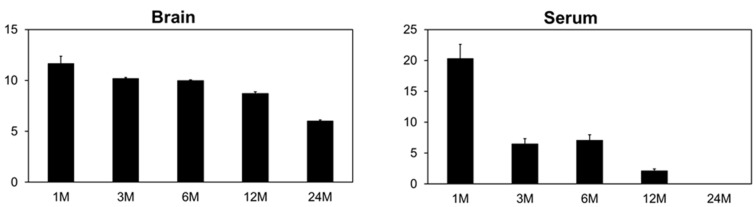

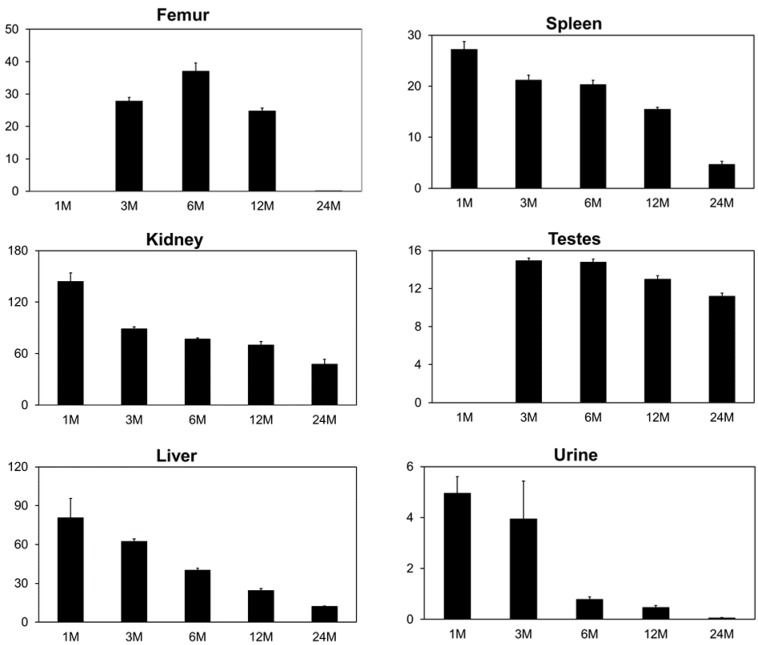
Tissue cobalt concentrations for WCoTa-implanted mice. The y-axis represents ng Co/gm tissue, except for “serum” (ng Co/mL serum) and “urine” (µg Co/mg creatinine). Data are the mean of 10 independent measurements. Error bars represent the standard error of the mean.

All metal analysis results can be found in the Supplemental Data section. The implanted pellets all solubilized to some extent and released the component metals. Over time, these metals were deposited in various tissues or excreted in the urine. Of the cobalt-containing compounds tested, only the WCoTa-implanted groups showed consistently significant increased levels of cobalt in the samples analyzed (Figure 4). The data presented in Figure 4 are all significantly higher than control values with the exception of the 24-month post-implantation time points for femur, kidney, spleen and urine, as well as the one-month time point for femur. For most samples, the highest cobalt concentrations are found at the one-month time point, with the levels dropping after that time. Surprisingly, elevated cobalt levels were found in the femur, an area where cobalt does not normally deposit [20].

**Figure 5:**
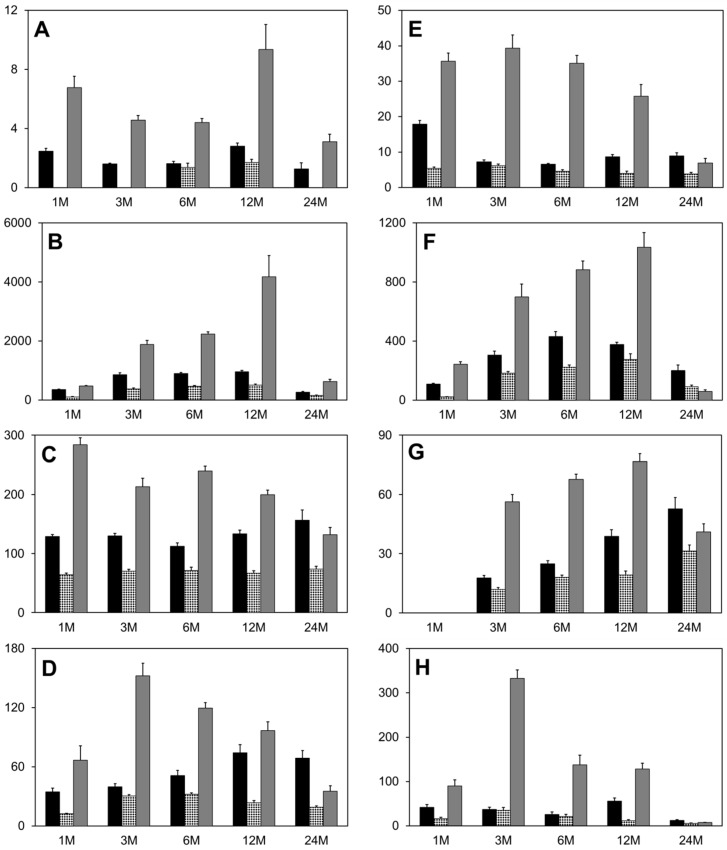
Tissue tungsten concentrations. (**A**) brain; (**B**) femur; (**C**) kidney; (**D**) liver; (**E**) serum; (**F**) spleen; (**G**) testes; (**H**) urine. The y-axis represents ng W/gm tissue, except for (E) (ng W/mL serum) and (H) (µg W/mg creatinine). Black bar, WTa group; stippled bar, WNiTa group; gray bar, WCoTa group. Data are the mean of 10 independent measurements. Error bars represent the standard error of the mean.

As with cobalt, all tissue tungsten levels depicted in Figure 5 are statistically higher than control values with the exception of the 1-, 3- and 24-month time points of the brain from the WNiTa-implanted group. In these cases, the tungsten levels were below the level of detection. Interestingly, despite all pellets containing the same percentage of tungsten, the WCoTa pellets consistently released more tungsten than did the WTa or WNiTa pellets. As noted above, the WCoTa pellets also released the greatest amount of cobalt when compared to other cobalt-containing pellets. Whether there is a synergistic effect of cobalt and tungsten with respect to the solubilization of these metals from the pellet is not yet known. Nickel results were inconsistent across time points, but the WNiTa-implanted group showed elevated levels of nickel in the kidney over time. Again, this raises the possibility of synergy between tungsten and nickel as the pellet begins to solubilize. Tantalum from the combination pellets also solubilized to a small extent and was found primarily in femur, kidney and spleen. Again, greater tantalum solubilization was seen from those pellets containing tungsten as opposed to the other formulations. Degradation of the tantalum control pellets was minimal, and any metal solubilized was primarily excreted in the urine.

To assess the degradation of the pellets at various times post-implantation, at euthanasia, the pellets were removed and their mass determined and compared to the pre-implantation values. As shown in Table 5, over the course of 24 months, tantalum pellets lost little of their original mass, while NiTa, CoTa and NiCoTa pellets lost a small amount (<5%) of the their pre-implantation mass. However, those pellets containing tungsten lost a significantly higher percentage of their mass as a result of implantation. After 24 months, the WTa and WNiTa pellets lost 25% and 23% of their initial pre-implantation mass, respectively. During the same time period, the WCoTa pellets lost approximately 65% of their initial mass, thus explaining the elevated metal content in the tissues collected from this group.

**Table 5:** Pellet recovery: percent of pre-implantation mass.

Group	Time Post-Implantation				
	1 M	3 M	6 M	12 M	24 M
Ta	98.7 ± 0.2	102.0 ± 1.1	97.9 ± 0.7	100.1 ± 0.2	101.9 ± 1.3
WTa	94.3 ± 3.0	92.6 ± 1.5	88.9 ± 1.2	79.8 ± 2.4	74.8 ± 4.8
NiTa	94.6 ± 1.2	94.0 ± 1.2	98.8 ± 1.0	96.6 ± 0.7	98.1 ± 1.0
CoTa	95.2 ± 1.0	94.0 ± 0.5	98.5 ± 0.4	97.2 ± 1.0	101.0 ± 0.6
WNiTa	97.2 ± 0.9	98.8 ± 1.4	92.8 ± 1.9	84.7 ± 1.6	77.2 ± 1.8
WCoTa	91.9 ± 1.0	86.2 ± 0.6	73.9 ± 1.2	48.4 ± 3.1	34.8 ± 2.1
NiCoTa	95.0 ± 0.5	97.0 ± 0.9	93.8 ± 0.7	94.7 ± 0.6	95.4 ± 0.7

Data represent the mass of the recovered implanted pellets normalized to the mass of the pellets pre-implantation. Data are expressed as the mean of six independent measurements (pellet recovery from six animals per group). Error represents the standard error of the mean.

## 4. Conclusions

Tungsten-based material has been increasingly used in a variety of applications, including in military munitions. One such formulation designed to replace depleted uranium in armor-penetrating munitions and comprised of tungsten, nickel and cobalt was shown to induce malignant rhabdomyosarcomas when implanted into the leg muscle of laboratory rats to simulate a shrapnel wound [11,12]. To ascertain if one component of this material could be responsible for the carcinogenic effect, pellets were manufactured with tantalum replacing each of the metals at an identical percentage. The resulting one- and two-metal composites were then implanted into the quadriceps muscle of B6C3F_1_ mice, and the mice were assessed over 24 months for any signs of adverse health effects. Implantation with WTa, CoTa or WNiTa pellets resulted in decreased survival, but not to the extent reported for WNiCo [13]. Implantation with CoTa, WTa and WCoTa pellets also resulted in sarcoma formation in a small number of mice, but far below the 80% reported for WNiCo-implanted mice [13]. This suggests that no single metal is solely responsible for the WNiCo-induced rhabdomyosarcomas and that a synergistic effect in tumor development is likely. Metal analysis data also demonstrated that pellets containing tungsten solubilized at a greater rate than pellets without tungsten. In fact, WCoTa pellets lost close to 65% of their mass after being implanted for 24 months, with the solubilized metals translocating to a variety of tissues, as well as being excreted in the urine. Of concern was the finding that these metals were capable of crossing the blood-brain and blood-testes barriers and localizing to the brain and testes, respectively. Degradation of the WCoTa pellets also resulted in increased content of all three metals in the femur, an area where cobalt is not normally found. This raises the possibility of a synergistic transport of tungsten and cobalt, as well as tantalum, to previously restricted tissues. Studies on soluble mixtures of tungsten, nickel and cobalt have demonstrated that they are capable of inducing reactive oxygen species and DNA damage in an *in vitro* model system [24], as well as inducing neoplastic changes in cultured human osteoblast cells in a synergistic manner [25]. In addition, particulate matter consisting of tungsten, nickel and cobalt has been shown to induce DNA strand breaks and cytotoxicity in both rat and human muscle cell lines, as well as result in changes in gene transcription [26]. Tungsten exposure has been shown to adversely affect B-cell development and alter the expression of genes involved in carcinogenesis [27,28,29]. Although once considered inert because of its perceived insoluble nature, recent studies have shown that embedded tungsten-containing materials can induce granulomas in humans [30,31]. In addition, tungsten particulates remaining in breast tissue from a radiation shield used for intraoperative radiotherapy in women with cancer were found to solubilize and be excreted in the urine for at least 20 months post-surgery [32]. Further research in this study found that, in a mouse breast cancer model, tungsten exposure enhanced metastasis by affecting the tumor microenvironment [32]. Together, these findings demonstrate our lack of understanding of the health effects of tungsten and tungsten-containing materials in the body and point out a need for further research in this area.

## Supplementary Materials

Supplementary File 1
