# Supplementary material for: The Role of the Component Metals in the Toxicity of Military-Grade Tungsten Alloy
Source: Toxics. 2015 Dec 8;3(4):499–514. doi: 10.3390/toxics3040499 (PMC5606641; doi:10.3390/toxics3040499)
Supplement: Supplementary File 1 [file toxics-03-00499-s001.pdf]

Supplementary Information

Table S1. Hematological parameters of 1-month implantation groups.

Group	WBC (10^3/μL)	RBC (10^6/μL)	HGB (g/dL)	HCT (%)	PLT (10^3/μL)	Lymphocytes 10^3/μL	Monocytes 10^3/μL	Granulocytes 10^3/μL
Sham	4.60 ± 0.35	9.42 ± 0.12	14.50 ± 0.16	45.48 ± 0.47	892.4 ± 5.9	2.76 ± 0.22	0.50 ± 0.05	1.34 ± 0.09
Ta	4.97 ± 0.96	8.54 ± 0.17	14.07 ± 0.27	41.51 ± 0.88 *	726.3 ± 51.1	2.34 ± 0.28	0.46 ± 0.06	2.17 ± 0.66
WTa	3.86 ± 0.52	9.28 ± 0.09	14.38 ± 0.20	41.15 ± 4.01	785.9 ± 85.5	1.90 ± 0.21 *	0.39 ± 0.05	1.57 ± 0.28
NiTa	6.13 ± 0.40 *	9.05 ± 0.13	14.13 ± 0.19	44.31 ± 0.62	963.2 ± 44.5	2.67 ± 0.21	0.62 ± 0.05	2.84 ± 0.23 *
CoTa	5.28 ± 0.65	8.53 ± 0.55	12.97 ± 0.91	41.33 ± 2.64	936.1 ± 69.8	2.18 ± 0.25	0.48 ± 0.06	2.62 ± 0.48
WNiTa	5.89 ± 0.90	9.31 ± 0.24	14.23 ± 0.36	44.13 ± 1.11	901.0 ± 69.0	2.24 ± 0.25	0.47 ± 0.06	3.18 ± 0.76
WCoTa	5.46 ± 0.51	9.03 ± 0.15	14.24 ± 0.23	43.77 ± 0.62	981.8 ± 42.1	2.48 ± 0.23	0.52 ± 0.05	2.46 ± 0.30 *
NiCoTa	5.37 ± 0.39	9.52 ± 0.16	15.09 ± 0.20	46.11 ± 0.77	868.8 ± 24.1	2.57 ± 0.22	0.49 ± 0.03	2.31 ± 0.19 *

Data represent the mean and standard error of the mean of 10 independent observations. An * indicates a result that is statistically different from the control (sham) at $p < 0.05$ using a one-way ANOVA followed by Dunnett's test for group mean comparisons. WBC, white blood cells; RBC, red blood cells; HGB, hemoglobin; HCT, hematocrit; PLT, platelets.

Table S2. Hematological parameters of 3-month implantation groups.

Group	WBC (10^3/μL)	RBC (10^6/μL)	HGB (g/dL)	HCT (%)	PLT (10^3/μL)	Lymphocytes 10^3/μL	Monocytes 10^3/μL	Granulocytes 10^3/μL
Sham	4.09 ± 0.51	8.60 ± 0.19	13.16 ± 0.25	40.55 ± 0.89	1070.1 ± 75.4	2.13 ± 0.31	0.22 ± 0.04	1.71 ± 0.28
Ta	4.29 ± 0.50	9.26 ± 0.13 *	14.13± 0.14 *	44.90 ± 0.65 *	864.3 ± 26.0 *	1.70 ± 0.07	0.34 ± 0.03 *	2.24 ± 0.48
WTa	3.79 ± 0.34	8.83 ± 0.15	13.69 ± 0.23	43.18 ± 0.75	805.9 ± 67.9 *	1.78 ± 0.22	0.38 ± 0.04 *	1.63 ± 0.19
NiTa	4.66 ± 0.40	8.87 ± 0.20	13.39 ± 0.29	43.11 ± 1.06	901.9 ± 32.1	1.97 ± 0.16	0.46 ± 0.04 *	2.23 ± 0.24
CoTa	5.03 ± 0.36	9.02 ± 0.21	14.03 ± 0.29	43.54 ± 1.02	1111.1 ± 58.2	2.33 ± 0.26	0.55 ± 0.04 *	2.15 ± 0.23
WNiTa	5.31 ± 0.49	9.19 ± 0.05	14.28 ± 0.10 *	44.22 ± 0.29 *	1006.3 ± 35.5	2.31 ± 0.20	0.49 ± 0.05 *	2.51 ± 0.35
WCoTa	5.16 ± 0.50	8.95 ± 0.09	14.17 ± 0.14 *	43.07 ± 0.47 *	1094.4 ± 43.9	1.70 ± 0.15	0.39 ± 0.03 *	3.07 ± 0.52
NiCoTa	5.13 ± 0.31	9.02 ± 0.14	13.97 ± 0.19 *	43.24 ± 0.73	1010.3 ± 49.3	2.40 ± 0.18	0.56 ± 0.05 *	2.19 ± 0.13

Data represent the mean and standard error of the mean of 10 independent observations. An * indicates a result that is statistically different from the control (sham) at $p < 0.05$ using a one-way ANOVA followed by Dunnett's test for group mean comparisons.

Table S3. Hematological parameters of 6-month implantation groups.

Group	WBC (10^3/µL)	RBC (10^6/µL)	HGB (g/dL)	HCT (%)	PLT (10^3/µL)	Lymphocytes 10^3/µL	Monocytes 10^3/µL	Granulocytes 10^3/µL
Sham	3.79 ± 0.36	8.46 ± 0.11	13.09 ± 0.16	39.75 ± 0.64	1159.2 ± 71.6	2.33 ± 0.30	0.19 ± 0.04	1.23 ± 0.12
Ta	4.86 ± 0.74	8.99 ± 0.11 *	13.77 ± 0.23	47.33 ± 0.67 *	988.4 ± 57.0	1.92 ± 0.29	0.34 ± 0.04 *	2.59 ± 0.77
WTa	4.71 ± 0.34	9.01 ± 0.15 *	13.88 ± 0.10 *	43.76 ± 0.38 *	967.4 ± 44.3	2.36 ± 0.24	0.49 ± 0.05 *	1.86 ± 0.10 *
NiTa	13.82 ± 8.30	8.50 ± 0.34	12.91 ± 0.56	40.69 ± 1.50	847.0 ± 138.6	1.77 ± 0.23	0.53 ± 0.14	11.53 ± 8.13
CoTa	5.98 ± 0.98	8.99 ± 0.11 *	13.62 ± 0.15	42.60 ± 0.49 *	1114.8 ± 35.1	2.26 ± 0.24	0.51 ± 0.05 *	3.21 ± 0.86
WNiTa	6.23 ± 0.96	8.75 ± 0.10	13.75 ± 0.17	41.98 ± 0.51 *	1091.7 ± 54.2	2.14 ± 0.22	0.44 ± 0.03 *	3.65 ± 1.01
WCoTa	4.11 ± 0.41	9.14 ± 0.16 *	14.34 ± 0.21 *	43.94 ± 0.76 *	990.0 ± 41.4	1.93 ± 0.27	0.39 ± 0.05 *	1.79 ± 0.14 *
NiCoTa	4.43 ± 0.29	9.00 ± 0.11 *	14.30 ± 0.11 *	43.26 ± 0.46 *	1021.6 ± 59.3	2.10 ± 0.15	0.40 ± 0.02 *	1.93 ± 0.15 *

Data represent the mean and standard error of the mean of 10 independent observations. An * indicates a result that is statistically different from the control (sham) at $p < 0.05$ using a one-way ANOVA followed by Dunnett's test for group mean comparisons.

Table S4. Hematological parameters of 12-month implantation groups.

Group	WBC (10^3/µL)	RBC (10^6/µL)	HGB (g/dL)	HCT (%)	PLT (10^3/µL)	Lymphocytes 10^3/µL	Monocytes 10^3/µL	Granulocytes 10^3/µL
Sham	6.65 ± 0.36	8.50 ± 0.26	13.89 ± 0.19	42.65 ± 0.48	1256.5 ± 60.2	3.45 ± 0.30	0.33 ± 0.05	1.55 ± 0.20
Ta	6.06 ± 0.96	8.89 ± 0.20	13.80 ± 0.27	42.57 ± 0.79	1205.4 ± 70.5	2.52 ± 0.34	0.43 ± 0.06	3.10 ± 0.65
WTa	5.23 ± 0.74	8.58 ± 0.70	12.95 ± 1.06	40.65 ± 3.47	1030.6± 34.0 *	2.67 ± 0.44	0.44 ± 0.08	2.12 ± 0.22
NiTa	5.19 ± 0.49	8.81 ± 0.10	13.20 ± 0.21	41.76 ± 0.61	990.7 ± 28.1 *	2.11 ± 0.19 *	0.34 ± 0.03	2.73 ± 0.35
CoTa	5.27 ± 0.61	8.28 ± 0.15	12.53 ± 0.20 *	39.31 ± 0.62 *	910.2 ± 20.3 *	1.79 ± 0.24 *	0.33 ± 0.04	3.14 ± 0.45 *
WNiTa	5.47 ± 0.39	9.24 ± 0.11 *	13.83 ± 0.19	44.00 ± 0.63	1048.3± 25.6 *	2.60 ± 0.27	0.48 ± 0.03 *	2.40 ± 0.11
WCoTa	5.66 ± 0.78	8.16 ± 0.77	12.50 ± 1.17	38.82 ± 3.72	929.0 ± 37.3 *	2.88 ± 0.43	0.48 ± 0.07	2.30 ± 0.29
NiCoTa	4.40 ± 0.60	8.82 ± 0.07	13.10 ± 0.17	41.10 ± 0.41	716.0 ± 55.1	2.30 ± 0.30	0.30 ± 0.06 *	1.80 ± 0.27

Data represent the mean and standard error of the mean of 10 independent observations. An * indicates a result that is statistically different from the control (sham) at $p < 0.05$ using a one-way ANOVA followed by Dunnett's test for group mean comparisons.

Table S5. Hematological parameters of 24-month implantation groups.

Group	WBC (10^3/µL)	RBC (10^6/µL)	HGB (g/dL)	HCT (%)	PLT (10^3/µL)	Lymphocytes 10^3/µL	Monocytes 10^3/µL	Granulocytes 10^3/µL
Sham	8.66 ± 1.00	8.47 ± 0.36	12.86 ± 0.52	39.18 ± 1.65	1124.1 ± 74.6	2.74 ± 0.43	0.58 ± 0.13	5.35 ± 0.85
Ta	7.16 ± 0.83	9.66 ± 0.49	13.89 ± 0.55	43.14 ± 1.87	1085.6 ± 72.4	2.19 ± 0.32	0.43 ± 0.06	4.53 ± 0.66
WTa	6.34 ± 0.38	8.72 ± 0.59	12.98 ± 0.72	39.83 ± 2.35	1146.5 ± 108.4	2.16 ± 0.23	0.39 ± 0.03	3.79 ± 0.32
NiTa	11.61 ± 4.99	8.74 ± 0.55	12.88 ± 0.54	39.68 ± 2.00	1154.7 ± 79.8	2.96 ± 0.47	0.54 ± 0.12	3.70 ± 0.57
CoTa	8.54 ± 1.27	8.90 ± 0.45	13.12 ± 0.58	40.58 ± 1.94	1103.5 ± 84.5	2.33 ± 0.25	0.42 ± 0.05	5.79 ± 1.23
WNiTa	6.93 ± 0.86	8.39 ± 0.36	13.09 ± 0.40	39.65 ± 1.31	1045.1 ± 77.4	2.64 ± 0.29	0.51 ± 0.07	3.78 ± 0.58
WCoTa	6.88 ± 0.95	8.62 ± 0.58	12.64 ± 0.61	39.31 ± 2.04	1038.4 ± 112.5	2.30 ± 0.31	0.96 ± 0.51	4.09 ± 0.73
NiCoTa	8.87 ± 1.48	7.87 ± 0.46	12.14 ± 0.67	37.57 ± 2.14	1011.5 ± 116.6	3.01 ± 0.62	0.62 ± 0.18	5.63 ± 0.90

Data represent the mean and standard error of the mean of 10 independent observations. An * indicates a result that is statistically different from the control (sham) at $p < 0.05$ using a one-way ANOVA followed by Dunnett's test for group mean comparisons.

Table S6. Organ body weight ratios from mice in 1-month implantation groups.

Group	Spleen/BW (mg/gm)	Kidney/BW (mg/gm)	Liver/BW (mg/gm)	Testes/BW (mg/gm)
Sham	2.63 ± 0.10	14.96 ± 0.20	46.54 ± 0.65	6.97 ± 0.24
Ta	2.92 ± 0.19	14.39 ± 0.23	51.05 ± 1.36 *	6.66 ± 0.14
WTa	2.82 ± 0.19	14.78 ± 0.23	47.72 ± 0.55	7.02 ± 0.14
NiTa	3.33 ± 0.23 *	14.89 ± 0.16	48.89 ± 0.87 *	6.27 ± 0.57
CoTa	4.13 ± 0.56 *	15.30 ± 0.22	49.39 ± 1.25 *	6.79 ± 0.22
WNiTa	3.15 ± 0.21	14.29 ± 0.21	49.70 ± 0.70	6.50 ± 0.26
WCoTa	3.51 ± 0.46	14.73 ± 0.40	49.27 ± 1.37 *	6.75 ± 0.25
NiCoTa	2.64 ± 0.09	14.30 ± 0.23	46.31 ± 0.38	6.31 ± 0.39

Data are the mean of 10 independent observations. Error the represents standard error of the mean. An * indicates a result that is statistically different from the control (sham) at $p < 0.05$ using one-way ANOVA followed by Dunnett's test for group mean comparisons.

Table S7. Organ body weight ratios from mice in 3-month implantation groups.

Group	Spleen/BW (mg/gm)	Kidney/BW (mg/gm)	Liver/BW (mg/gm)	Testes/BW (mg/gm)
Sham	3.02 ± 0.23	13.44 ± 0.34	45.16 ± 0.69	6.52 ± 0.15
Ta	2.55 ± 0.19	13.25 ± 0.24	47.14 ± 1.04	5.96 ± 0.08 *
WTa	2.49 ± 0.14	13.08 ± 0.28	46.73 ± 1.04	5.82 ± 0.14 *
NiTa	3.01 ± 0.24	14.09 ± 0.31	49.43 ± 1.52 *	5.88 ± 0.1 *
CoTa	3.21 ± 0.23	13.65 ± 0.31	49.41 ± 1.03 *	6.13 ± 0.33
WNiTa	2.64 ± 0.15	12.82 ± 0.17	43.47 ± 4.24	5.89 ± 0.09 *
WCoTa	3.57 ± 0.32	14.25 ± 0.19	47.43 ± 0.98	5.97 ± 0.11 *
NiCoTa	2.43 ± 0.10	12.99 ± 0.30	45.94 ± 0.75	6.17 ± 0.24

Data are the mean of 10 independent observations. Error represents the standard error of the mean. An * indicates a result that is statistically different from the control (sham) at $p < 0.05$ using one-way ANOVA followed by Dunnett's test for group mean comparisons.

Table S8. Organ body weight ratios from mice in 6-month implantation groups.

Group	Spleen/BW (mg/gm)	Kidney/BW (mg/gm)	Liver/BW (mg/gm)	Testes/BW (mg/gm)
Sham	2.44 ± 0.19	12.62 ± 0.31	41.84 ± 1.22	5.56 ± 0.15
Ta	2.85 ± 0.28	16.37 ± 3.32	43.96 ± 0.75	5.39 ± 0.15
WTa	2.58 ± 0.14	13.98 ± 0.41 *	46.38 ± 0.69 *	5.67 ± 0.17
NiTa	2.47 ± 0.20	14.18 ± 0.67	49.28 ± 2.61	5.72 ± 0.12
CoTa	3.10 ± 0.40	13.57 ± 0.26	47.23 ± 0.96 *	5.56 ± 0.14
WNiTa	2.72 ± 0.24	13.75 ± 0.29 *	46.90 ± 1.45 *	5.51 ± 0.14
WCoTa	2.02 ± 0.06	12.86 ± 0.39	41.19 ± 1.04	5.48 ± 0.13
NiCoTa	2.50 ± 0.17	12.97 ± 0.22	47.91 ± 1.32 *	5.10 ± 0.08

Data are the mean of 10 independent observations. Error represents the standard error of the mean. An * indicates a result that is statistically different from the control (sham) at $p < 0.05$ using one-way ANOVA followed by Dunnett's test for group mean comparisons.

Table S9. Organ body weight ratios from mice in 12-month implantation groups.

Group	Spleen/BW (mg/gm)	Kidney/BW (mg/gm)	Liver/BW (mg/gm)	Testes/BW (mg/gm)
Sham	2.52 ± 0.12	13.28 ± 0.29	44.54 ± 1.35	4.88 ± 0.09
Ta	2.77 ± 0.24	13.52 ± 0.35	43.80 ± 0.86	4.66 ± 0.16
WTa	2.22 ± 0.25	13.42 ± 0.56	43.62 ± 1.34	4.48 ± 0.13 *
NiTa	3.82 ± 1.45	13.69 ± 0.29	45.08 ± 0.79	5.33 ± 0.15 *
CoTa	2.33 ± 0.14	13.59 ± 0.45	43.12 ± 1.16	4.99 ± 0.18
WNiTa	2.40 ± 0.15	13.36 ± 0.41	46.31 ± 1.35	4.83 ± 0.14
WCoTa	2.23 ± 0.10	13.53 ± 0.43	45.15 ± 1.11	5.07 ± 0.11
NiCoTa	2.29 ± 0.08	13.27 ± 0.28	44.09 ± 0.63	4.37 ± 0.33

Data are the mean of 10 independent observations. Error represents the standard error of the mean. An * indicates a result that is statistically different from the control (sham) at $p < 0.05$ using one-way ANOVA followed by Dunnett's test for group mean comparisons.

Table S10. Organ body weight ratios from mice in 24-month implantation groups.

Group	Spleen/BW (mg/gm)	Kidney/BW (mg/gm)	Liver/BW (mg/gm)	Testes/BW (mg/gm)
Sham	6.25 ± 1.89	12.77 ± 0.41	52.92 ± 4.51	4.43 ± 0.10
Ta	9.09 ± 2.27	15.44 ± 0.80 *	60.91 ± 4.11	4.59 ± 0.13
WTa	9.57 ± 5.62	14.06 ± 0.29	59.87 ± 8.50	4.91 ± 0.15 *
NiTa	5.25 ± 1.20	13.66 ± 0.36	63.90 ± 8.37	4.72 ± 0.13
CoTa	7.38 ± 1.52	13.94 ± 0.27 *	57.69 ± 5.87	4.70 ± 0.16
WNiTa	3.76 ± 0.51	13.32 ± 0.48	47.35 ± 3.72	4.67 ± 0.18
WCoTa	10.92 ± 4.67	13.72 ± 0.35	60.20 ± 6.43	4.78 ± 0.16
NiCoTa	6.95 ± 2.03	13.72 ± 0.27	53.25 ± 3.59	4.94 ± 0.16 *

Data are the mean of 20 independent observations. Error represents the standard error of the mean. An * indicates a result that is statistically different from the control (sham) at $p < 0.05$ using one-way ANOVA followed by Dunnett's test for group mean comparisons.

Table S11. Brain metal levels.

**1-Month Groups**				
Group	Cobalt	Nickel	Tantalum	Tungsten
Control	2.32 ± 0.15	BD	BD	BD
Ta	2.30 ± 0.12	BD	BD	BD
WTa	1.88 ± 0.06	BD	BD	2.48 ± 0.18 *
NiTa	2.05 ± 0.05	BD	BD	BD
CoTa	2.46 ± 0.16	BD	BD	BD
WNiTa	1.79 ± 0.07	BD	BD	BD
WCoTa	11.65 ± 0.72 *	BD	BD	6.78 ± 0.77 *
NiCoTa	2.84 ± 0.20	10.87 ± 0.79 *	BD	BD
**3-Month Groups**				
Group	Cobalt	Nickel	Tantalum	Tungsten
Control	1.90 ± 0.04	BD	BD	BD
Ta	1.92 ± 0.02	BD	BD	BD
WTa	1.90 ± 0.02	BD	BD	1.62 ± 0.05 *
NiTa	1.95 ± 0.02	BD	BD	BD
CoTa	1.95 ± 0.01	BD	BD	BD
WNiTa	1.99 ± 0.01	BD	BD	BD
WCoTa	10.19 ± 0.10 *	BD	BD	4.58 ± 0.31 *
NiCoTa	2.06 ± 0.01	BD	BD	BD
**6-Month Groups**				
Group	Cobalt	Nickel	Tantalum	Tungsten
Control	1.85 ± 0.02	BD	BD	BD
Ta	1.80 ± 0.01	BD	BD	BD
WTa	2.41 ± 0.02	BD	BD	1.63 ± 0.16 *
NiTa	2.32 ± 0.02	BD	BD	BD
CoTa	2.42 ± 0.03	BD	BD	BD
WNiTa	2.23 ± 0.03	3.91 ± 0.74 *	BD	1.36 ± 0.31 *
WCoTa	9.98 ± 0.08 *	4.38 ± 0.91 *	BD	4.42 ± 0.27 *
NiCoTa	2.32 ± 0.04	BD	BD	BD

Table S11. *Cont.*

12-Month Groups				
Group	Cobalt	Nickel	Tantalum	Tungsten
Control	1.93 ± 0.03	BD	BD	BD
Ta	2.04 ± 0.09	BD	BD	BD
WTa	2.12 ± 0.04	BD	BD	2.82 ± 0.21 *
NiTa	2.11 ± 0.01	1.39 ± 0.26	BD	BD
CoTa	2.16 ± 0.05	BD	BD	BD
WNiTa	2.40 ± 0.05	BD	BD	1.69 ± 0.24 *
WCoTa	8.71 ± 0.16 *	BD	BD	9.36 ± 1.68 *
NiCoTa	2.25 ± 0.02	10.12 ± 1.53 *	BD	BD
**24-Month Groups**				
Group	Cobalt	Nickel	Tantalum	Tungsten
Control	2.92 ± 0.13	BD	BD	BD
Ta	2.80 ± 0.06	BD	BD	BD
WTa	3.04 ± 0.09	BD	BD	1.27 ± 0.42 *
NiTa	2.67 ± 0.08	BD	BD	BD
CoTa	2.75 ± 0.11	BD	BD	BD
WNiTa	2.84 ± 0.05	BD	BD	BD
WCoTa	6.02 ± 0.09 *	BD	BD	3.12 ± 0.50 *
NiCoTa	2.90 ± 0.07	BD	BD	BD

All mice were implanted with 4 pellets of test metal. Data represent the mean and standard error of the mean of 10 independent measurements and are expressed as ng metal per g tissue. An * indicates a result that is statistically different from the control (sham) at $p < 0.05$ using one-way ANOVA. BD, below the limit of detection.

Table S12. Femur metal levels.

1-Month Groups				
Group	Cobalt	Nickel	Tantalum	Tungsten
Control	BD	BD	BD	BD
Ta	BD	BD	BD	BD
WTa	BD	BD	2.46 ± 0.36 *	357.70 ± 9.43 *
NiTa	BD	BD	BD	BD
CoTa	BD	BD	BD	BD
WNiTa	BD	BD	5.40 ± 0.75 *	111.17 ± 5.83 *
WCoTa	BD	BD	12.28 ± 1.11 *	479.13 ± 13.09 *
NiCoTa	BD	BD	BD	BD
**3-Month Groups**				
Group	Cobalt	Nickel	Tantalum	Tungsten
Control	BD	BD	BD	BD
Ta	BD	BD	17.02 ± 4.60 *	BD
WTa	BD	BD	44.78 ± 2.60 *	862.49 ± 59.45 *
NiTa	BD	BD	27.87 ± 2.35 *	BD
CoTa	BD	BD	11.62 ± 1.27 *	BD
WNiTa	BD	BD	24.45 ± 1.76 *	381.07 ± 29.72 *
WCoTa	27.83 ± 1.12 *	BD	112.84 ± 5.19 *	1887.68± 133.50 *
NiCoTa	BD	BD	7.20 ± 0.25 *	BD

Table S12. *Cont.*

6-Month Groups				
Group	Cobalt	Nickel	Tantalum	Tungsten
Control	BD	BD	BD	BD
Ta	BD	BD	BD	BD
WTa	BD	BD	56.14 ± 3.67 *	898.67 ± 38.95 *
NiTa	BD	BD	30.50 ± 1.39 *	BD
CoTa	BD	BD	8.36 ± 0.57 *	BD
WNiTa	BD	BD	32.94 ± 2.68 *	467.50 ± 22.32 *
WCoTa	37.07 ± 2.49 *	BD	161.83 ± 10.71 *	2239.09 ± 76.62 *
NiCoTa	BD	BD	6.46 ± 0.63 *	BD
**12-Month Groups**				
Group	Cobalt	Nickel	Tantalum	Tungsten
Control	BD	BD	BD	BD
Ta	BD	BD	BD	BD
WTa	BD	BD	26.67 ± 2.55 *	956.73 ± 51.26 *
NiTa	BD	BD	55.81 ± 8.90 *	BD
CoTa	BD	BD	43.45 ± 4.96 *	BD
WNiTa	BD	BD	50.33 ± 3.99 *	506.84 ± 38.48 *
WCoTa	24.74 ± 0.91 *	BD	233.55 ± 27.93 *	4174.36 ± 718.84 *
NiCoTa	BD	BD	7.39 ± 0.13 *	BD
**24-Month Groups**				
Group	Cobalt	Nickel	Tantalum	Tungsten
Control	BD	BD	BD	BD
Ta	BD	BD	2.81 ± 0.50 *	BD
WTa	BD	BD	16.15 ± 2.07 *	267.56 ± 27.34 *
NiTa	BD	BD	7.82 ± 1.69 *	BD
CoTa	BD	BD	3.01 ± 0.46 *	BD
WNiTa	BD	BD	66.63 ± 8.75 *	156.84 ± 12.05 *
WCoTa	BD	BD	142.32 ± 17.04 *	631.02 ± 68.09 *
NiCoTa	BD	BD	2.22 ± 0.42 *	BD

All mice were implanted with 4 pellets of test metal. Data represent the mean and standard error of the mean of 10 independent measurements and are expressed as ng metal per g tissue. An * indicates a result that is statistically different from the control (sham) at $p < 0.05$ using one-way ANOVA. BD, below the limit of detection.

Table S13. Kidney metal levels.

1-Month Groups				
Group	Cobalt	Nickel	Tantalum	Tungsten
Control	36.44 ± 0.90	4.27 ± 0.97	2.20 ± 0.15	BD
Ta	31.83 ± 0.86	1.77 ± 0.99	2.00 ± 0.13	BD
WTa	54.51 ± 1.17 *	8.31 ± 1.81	4.18 ± 0.20 *	128.68 ± 3.50 *
NiTa	49.02 ± 1.27 *	4.69 ± 0.60	3.78 ± 0.19 *	BD
CoTa	42.33 ± 2.49 *	3.97 ± 0.68	2.81 ± 0.18 *	BD
WNiTa	40.08 ± 1.01 *	121.69 ± 5.50 *	BD	64.13 ± 2.82 *
WCoTa	144.00 ± 10.17 *	11.79 ± 0.99 *	1.68 ± 0.25	283.91 ± 11.58 *
NiCoTa	53.73 ± 2.04 *	10.49 ± 0.85 *	BD	BD

Table S13. *Cont.*

3-Month Groups				
Group	Cobalt	Nickel	Tantalum	Tungsten
Control	42.67 ± 0.79	6.26 ± 0.39	BD	BD
Ta	50.95 ± 1.01 *	5.10 ± 0.50 *	BD	BD
WTa	43.74 ± 1.22	11.91 ± 0.50 *	4.16 ± 0.24 *	129.69 ± 4.26 *
NiTa	39.38 ± 0.75	10.15 ± 0.34 *	2.22 ± 0.13 *	BD
CoTa	40.08 ± 1.44	11.67 ± 1.21 *	BD	BD
WNiTa	44.63 ± 0.71	28.54 ± 2.64 *	2.15 ± 0.24 *	70.42 ± 2.92 *
WCoTa	88.98 ± 2.07 *	5.01 ± 0.72	12.48 ± 0.72 *	213.25 ± 14.13 *
NiCoTa	39.72 ± 0.89	7.78 ± 0.97	BD	BD
**6-Month Groups**				
Group	Cobalt	Nickel	Tantalum	Tungsten
Control	45.70 ± 1.66	3.06 ± 0.18	BD	BD
Ta	44.27 ± 1.72	2.21 ± 0.09 *	BD	BD
WTa	43.42 ± 2.14	8.32 ± 0.24 *	2.17 ± 0.23 *	112.20 ± 5.84 *
NiTa	38.35 ± 0.48	9.92 ± 0.14 *	BD	BD
CoTa	38.48 ± 1.29	10.86 ± 0.88 *	BD	BD
WNiTa	41.81 ± 1.43	13.46 ± 1.08 *	1.59 ± 0.29	71.45 ± 5.40 *
WCoTa	77.02 ± 1.24 *	5.81 ± 0.37 *	6.60 ± 1.08 *	239.63 ± 8.51 *
NiCoTa	40.53 ± 2.23	6.11 ± 0.54 *	BD	BD
**12-Month Groups**				
Group	Cobalt	Nickel	Tantalum	Tungsten
Control	36.26 ± 0.63	5.33 ± 0.23	BD	BD
Ta	33.05 ± 1.32	4.40 ± 0.32	BD	BD
WTa	30.61 ± 1.24 *	4.68 ± 0.45	BD	133.17 ± 6.41 *
NiTa	42.22 ± 0.74 *	5.62 ± 0.71	BD	BD
CoTa	41.79 ± 1.47 *	4.79 ± 0.22	BD	BD
WNiTa	12.14 ± 0.62 *	3.31 ± 0.34 *	3.76 ± 0.49 *	66.68 ± 4.11 *
WCoTa	70.00 ± 4.20 *	4.87 ± 0.50	13.93 ± 1.63 *	199.45 ± 7.81 *
NiCoTa	58.41 ± 35.54 *	3.04 ± 0.80 *	BD	BD
**24-Month Groups**				
Group	Cobalt	Nickel	Tantalum	Tungsten
Control	35.09 ± 5.41	23.87 ± 1.95	BD	BD
Ta	43.73 ± 1.69	21.34 ± 1.65	BD	BD
WTa	42.51 ± 1.80	17.49 ± 1.79 *	BD	156.63 ± 17.27 *
NiTa	42.51 ± 2.83	12.86 ± 1.45 *	BD	BD
CoTa	46.33 ± 2.85	22.50 ± 2.41	BD	BD
WNiTa	46.43 ± 2.58	21.18 ± 1.27	1.59 ± 0.21	73.78 ± 4.43 *
WCoTa	47.68 ± 5.68	14.46 ± 1.33 *	2.71 ± 0.49 *	131.86 ± 12.14 *
NiCoTa	35.07 ± 5.34	11.59 ± 0.82 *	BD	BD

All mice were implanted with 4 pellets of test metal. Data represent the mean and standard error of the mean of 10 independent measurements and are expressed as ng metal per g tissue. An * indicates a result that is statistically different from the control (sham) at $p < 0.05$ using one-way ANOVA. BD, below the limit of detection.

Table S14. Liver metal levels.

Group	Cobalt	Nickel	Tantalum	Tungsten
**1-Month Groups**				
Group	Cobalt	Nickel	Tantalum	Tungsten
Control	29.57 ± 1.73	17.00 ± 3.31	BD	2.66 ± 0.68
Ta	25.49 ± 3.53	24.95 ± 8.68	BD	BD
WTa	9.23 ± 0.74 *	13.22 ± 1.69	BD	34.90 ± 3.56 *
NiTa	8.02 ± 0.59 *	17.05 ± 2.42	BD	BD
CoTa	8.13 ± 0.35 *	25.97 ± 2.55	BD	BD
WNiTa	9.39 ± 0.32 *	39.13 ± 4.64 *	BD	12.14 ± 0.80 *
WCoTa	80.74 ± 14.85 *	63.49 ± 4.96 *	BD	66.86 ± 14.51 *
NiCoTa	19.09 ± 1.71 *	148.32 ± 15.56 *	BD	BD
**3-Month Groups**				
Group	Cobalt	Nickel	Tantalum	Tungsten
Control	11.12 ± 0.25	8.38 ± 1.09	BD	BD
Ta	9.26 ± 0.19	6.20 ± 0.80 *	BD	BD
WTa	8.00 ± 0.25 *	16.00 ± 1.48 *	BD	39.89 ± 2.88 *
NiTa	7.51 ± 0.18 *	2.82 ± 0.26 *	BD	BD
CoTa	8.37 ± 0.30 *	5.34 ± 1.14 *	BD	BD
WNiTa	10.12 ± 0.11	20.58 ± 1.67 *	BD	30.30 ± 1.49 *
WCoTa	62.36 ± 1.90 *	16.28 ± 0.72 *	3.77 ± 0.80 *	152.15 ± 12.79 *
NiCoTa	11.90 ± 0.28	23.80 ± 1.86 *	BD	BD
**6-Month Groups**				
Group	Cobalt	Nickel	Tantalum	Tungsten
Control	9.83 ± 0.22	3.62 ± 0.49	BD	BD
Ta	8.77 ± 0.12	2.31 ± 0.06	BD	BD
WTa	8.34 ± 0.09	4.47 ± 0.46	2.12 ± 0.41	51.03 ± 5.35 *
NiTa	8.77 ± 0.15	5.12 ± 0.62	BD	BD
CoTa	8.27 ± 0.29	18.39 ± 2.87 *	BD	BD
WNiTa	9.23 ± 0.25	6.46 ± 0.54 *	BD	32.00 ± 1.48 *
WCoTa	40.10 ± 1.50 *	3.89 ± 0.46	16.09 ± 2.26 *	119.66 ± 5.47 *
NiCoTa	8.52 ± 0.13	3.73 ± 0.14	BD	BD
**12-Month Groups**				
Group	Cobalt	Nickel	Tantalum	Tungsten
Control	7.03 ± 0.20	6.76 ± 0.48	BD	BD
Ta	8.22 ± 0.20 *	7.90 ± 0.99	BD	BD
WTa	8.48 ± 0.19 *	9.07 ± 0.88	3.09 ± 0.47 *	74.36 ± 8.22 *
NiTa	9.16 ± 0.27 *	12.62 ± 1.22 *	7.00 ± 0.40 *	BD
CoTa	9.22 ± 0.11 *	12.17 ± 0.83 *	3.78 ± 0.20	BD
WNiTa	8.69 ± 0.20 *	8.39 ± 1.17	23.03 ± 2.76 *	23.35 ± 2.42 *
WCoTa	24.54 ± 1.34 *	7.57 ± 0.91	74.83 ± 4.97 *	96.69 ± 8.90 *
NiCoTa	8.77 ± 0.15 *	8.58 ± 1.03	BD	BD

Table S14. *Cont.*

24-Month Groups				
Group	Cobalt	Nickel	Tantalum	Tungsten
Control	8.62 ± 0.27	37.52 ± 1.53	BD	BD
Ta	9.77 ± 0.16 *	45.25 ± 4.85	BD	BD
WTa	9.39 ± 0.14 *	38.52 ± 5.59	2.47 ± 0.65	69.02 ± 7.51 *
NiTa	8.95 ± 0.42	19.57 ± 2.11 *	BD	BD
CoTa	10.33 ± 0.27 *	27.47 ± 3.96 *	BD	BD
WNiTa	8.52 ± 0.31	14.92 ± 1.43 *	3.33 ± 0.70 *	18.92 ± 1.57 *
WCoTa	12.15 ± 0.41 *	19.77 ± 2.90 *	14.15 ± 1.82 *	35.34 ± 5.58 *
NiCoTa	9.81 ± 0.45 *	19.89 ± 1.15 *	BD	BD

All mice were implanted with 4 pellets of test metal. Data represent the mean and standard error of the mean of 10 independent measurements and are expressed as ng metal per g tissue. An * indicates a result that is statistically different from the control (sham) at $p < 0.05$ using one-way ANOVA. BD, below the limit of detection.

Table S15. Serum metal levels.

1-Month Groups				
Group	Cobalt	Nickel	Tantalum	Tungsten
Control	BD	BD	BD	BD
Ta	BD	18.44 ± 1.94 *	BD	BD
WTa	BD	12.91 ± 2.95 *	BD	17.88 ± 1.07 *
NiTa	BD	11.20 ± 2.19 *	BD	BD
CoTa	BD	8.44 ± 0.92 *	BD	BD
WNiTa	BD	18.83 ± 1.05 *	BD	5.44 ± 0.36 *
WCoTa	20.30 ± 2.29 *	5.02 ± 1.32 *	BD	35.65 ± 2.34 *
NiCoTa	BD	BD	BD	BD
**3-Month Groups**				
Group	Cobalt	Nickel	Tantalum	Tungsten
Control	BD	BD	BD	BD
Ta	BD	BD	BD	BD
WTa	BD	BD	BD	7.23 ± 0.59 *
NiTa	BD	3.82 ± 0.82 *	BD	BD
CoTa	BD	4.73 ± 1.09 *	BD	BD
WNiTa	BD	4.00 ± 0.29 *	BD	6.19 ± 0.42 *
WCoTa	6.46 ± 0.89 *	4.72 ± 0.56 *	BD	39.32 ± 3.72 *
NiCoTa	BD	7.37 ± 1.69 *	BD	BD
**6-Month Groups**				
Group	Cobalt	Nickel	Tantalum	Tungsten
Control	BD	BD	BD	BD
Ta	BD	BD	BD	BD
WTa	BD	2.33 ± 0.14	BD	6.59 ± 0.22 *
NiTa	BD	3.20 ± 0.31 *	BD	BD
CoTa	BD	1.85 ± 0.22	BD	BD
WNiTa	BD	1.72 ± 0.21	BD	4.57 ± 0.43 *
WCoTa	7.04 ± 0.91 *	2.44 ± 0.19	BD	35.06 ± 2.21 *
NiCoTa	BD	2.22 ± 0.12	BD	BD

Table S15. *Cont.*

12-Month Groups				
Group	Cobalt	Nickel	Tantalum	Tungsten
Control	BD	7.33 ± 0.54	BD	BD
Ta	BD	9.26 ± 1.43	BD	BD
WTa	BD	6.97 ± 1.58	BD	8.70 ± 0.55 *
NiTa	BD	3.01 ± 0.84 *	BD	BD
CoTa	BD	2.26 ± 0.50 *	BD	BD
WNiTa	BD	BD	BD	4.03 ± 0.57 *
WCoTa	2.13 ± 0.28	BD	BD	25.76 ± 3.29 *
NiCoTa	BD	BD	BD	BD
**24-Month Groups**				
Group	Cobalt	Nickel	Tantalum	Tungsten
Control	BD	26.09 ± 3.87	BD	BD
Ta	BD	21.14 ± 3.08	BD	BD
WTa	BD	14.75 ± 1.74 *	BD	8.92 ± 0.89 *
NiTa	BD	16.46 ± 1.62 *	BD	BD
CoTa	BD	8.41 ± 0.83 *	BD	BD
WNiTa	BD	10.47 ± 1.60 *	BD	3.80 ± 0.50 *
WCoTa	BD	15.35 ± 2.00 *	BD	6.90 ± 1.34 *
NiCoTa	BD	8.46 ± 0.93 *	BD	BD

All mice were implanted with 4 pellets of test metal. Data represent the mean and standard error of the mean of 10 independent measurements and are expressed as ng metal per ml serum. An * indicates a result that is statistically different from the control (sham) at $p < 0.05$ using one-way ANOVA. BD, below the limit of detection.

Table S16. Spleen metal levels.

1-Month Groups				
Group	Cobalt	Nickel	Tantalum	Tungsten
Control	3.04 ± 0.29	51.66 ± 8.49	BD	BD
Ta	2.65 ± 0.12	41.64 ± 5.77	BD	BD
WTa	2.11 ± 0.16	BD	BD	109.61 ± 3.84 *
NiTa	2.25 ± 0.14	6.49 ± 2.41 *	BD	BD
CoTa	2.96 ± 0.57	BD	BD	BD
WNiTa	2.25 ± 0.09	24.55 ± 3.50 *	BD	21.42 ± 2.03 *
WCoTa	27.21 ± 1.56 *	22.79 ± 3.89 *	BD	243.32 ± 15.83
NiCoTa	11.40 ± 1.19 *	211.09 ± 22.86 *	1.83 ± 0.49	BD
**3-Month Groups**				
Group	Cobalt	Nickel	Tantalum	Tungsten
Control	2.54 ± 0.25	29.88 ± 4.31	BD	BD
Ta	2.37 ± 0.09	36.19 ± 6.55	BD	BD
WTa	3.15 ± 0.08 *	122.63 ± 19.72 *	7.19 ± 0.58 *	305.43 ± 27.63 *
NiTa	2.92 ± 0.17	79.13 ± 18.03 *	4.60 ± 0.37 *	BD
CoTa	2.97 ± 0.12	70.88 ± 8.45 *	BD	BD
WNiTa	3.14 ± 0.20	115.32 ± 17.44 *	6.36 ± 0.86 *	182.61 ± 11.82 *
WCoTa	21.21 ± 0.94 *	55.40 ± 11.94	19.42 ± 2.26 *	699.35 ± 86.11 *
NiCoTa	3.29 ± 0.16 *	133.28 ± 11.66 *	BD	BD

Table S16. *Cont.*

6-Month Groups				
Group	Cobalt	Nickel	Tantalum	Tungsten
Control	2.48 ± 0.17	15.28 ± 2.55	BD	BD
Ta	2.53 ± 0.32	12.09 ± 2.17	1.85 ± 0.33	BD
WTa	3.66 ± 0.06 *	110.82 ± 26.72 *	16.33 ± 1.51 *	431.77 ± 32.83 *
NiTa	3.13 ± 0.14 *	46.41 ± 9.52 *	8.46 ± 0.99 *	BD
CoTa	2.78 ± 0.09	60.76 ± 9.50 *	2.24 ± 0.18 *	BD
WNiTa	3.63 ± 0.40 *	120.67 ± 22.39 *	9.40 ± 1.62 *	221.49 ± 17.57 *
WCoTa	20.32 ± 0.85 *	124.26 ± 18.70 *	41.03 ± 2.12 *	882.81 ± 59.11 *
NiCoTa	4.83 ± 0.41	125.43 ± 22.80 *	BD	BD
**12-Month Groups**				
Group	Cobalt	Nickel	Tantalum	Tungsten
Control	3.10 ± 0.23	45.47 ± 5.70	BD	BD
Ta	2.86 ± 0.25	25.65 ± 3.59 *	BD	BD
WTa	4.86 ± 0.51 *	87.81 ± 20.75	2.03 ± 0.07	376.88 ± 15.52 *
NiTa	2.76 ± 0.11	71.10 ± 19.80	10.42 ± 0.81 *	BD
CoTa	2.83 ± 0.15	35.85 ± 6.84	5.33 ± 0.53 *	BD
WNiTa	3.09 ± 0.10	81.49 ± 19.84	26.71 ± 3.38 *	273.75 ± 40.85 *
WCoTa	15.49 ± 0.36 *	59.22 ± 8.29	79.20 ± 9.08 *	1035.43 ± 98.79 *
NiCoTa	3.33 ± 0.15	84.70 ± 17.66 *	1.87 ± 0.19	BD
**24-Month Groups**				
Group	Cobalt	Nickel	Tantalum	Tungsten
Control	3.39 ± 0.29	63.03 ± 5.92	BD	BD
Ta	3.11 ± 0.26	70.37 ± 9.20	BD	BD
WTa	2.50 ± 0.12	68.81 ± 12.35	BD	200.22 ± 38.57 *
NiTa	2.56 ± 0.40	44.65 ± 6.74	BD	BD
CoTa	2.24 ± 0.19	62.99 ± 10.18	BD	BD
WNiTa	2.71 ± 0.19	113.71 ± 14.44 *	BD	90.57 ± 11.08 *
WCoTa	4.68 ± 0.58	59.95 ± 8.50	BD	58.20 ± 11.45 *
NiCoTa	2.58 ± 0.05	40.99 ± 3.74 *	BD	BD

All mice were implanted with 4 pellets of test metal. Data represent the mean and standard error of the mean of 10 independent measurements and are expressed as ng metal per g tissue. An * indicates a result that is statistically different from the control (sham) at $p < 0.05$ using one-way ANOVA. BD, below the limit of detection.

Table S17. Testes metal levels.

1-Month Groups				
Group	Cobalt	Nickel	Tantalum	Tungsten
Control	ND	ND	ND	ND
Ta	ND	ND	ND	ND
WTa	ND	ND	ND	ND
NiTa	ND	ND	ND	ND
CoTa	ND	ND	ND	ND
WNiTa	ND	ND	ND	ND
WCoTa	ND	ND	ND	ND
NiCoTa	ND	ND	ND	ND

Table S17. *Cont.*

3-Month Groups				
Group	Cobalt	Nickel	Tantalum	Tungsten
Control	5.10 ± 0.12	7.07 ± 0.55	BD	BD
Ta	4.55 ± 0.06	5.86 ± 0.83	BD	BD
WTa	5.32 ± 0.11	20.66 ± 6.38	3.94 ± 0.44 *	17.63 ± 1.35 *
NiTa	5.44 ± 0.05	26.93 ± 5.14 *	3.23 ± 0.19 *	BD
CoTa	5.24 ± 0.07	22.12 ± 5.10 *	BD	BD
WNiTa	5.40 ± 0.09	34.62 ± 4.89 *	4.82 ± 0.35 *	12.00 ± 0.80 *
WCoTa	14.92 ± 0.29 *	28.88 ± 6.62 *	16.65 ± 1.68 *	56.23 ± 3.70 *
NiCoTa	5.22 ± 0.06	20.78 ± 5.17 *	BD	BD
**6-Month Groups**				
Group	Cobalt	Nickel	Tantalum	Tungsten
Control	4.92 ± 0.15	3.38 ± 0.32	BD	BD
Ta	5.36 ± 0.11	3.05 ± 0.17	BD	BD
WTa	5.52 ± 0.09	7.85 ± 1.77 *	10.72 ± 0.53 *	24.83 ± 1.61 *
NiTa	5.71 ± 0.12	7.53 ± 2.21	3.35 ± 0.32 *	BD
CoTa	5.04 ± 0.10	21.48 ± 4.16 *	BD	BD
WNiTa	5.81 ± 0.14	14.94 ± 3.45 *	5.84 ± 0.77 *	18.22 ± 0.83 *
WCoTa	14.78 ± 0.34 *	19.51 ± 5.41 *	19.67 ± 3.79 *	67.68 ± 2.65 *
NiCoTa	5.67 ± 0.18	25.25 ± 6.79 *	BD	BD
**12-Month Groups**				
Group	Cobalt	Nickel	Tantalum	Tungsten
Control	5.62 ± 0.19	11.85 ± 0.54	BD	BD
Ta	5.57 ± 0.20	17.30 ± 2.80	BD	BD
WTa	6.49 ± 0.20	13.51 ± 1.70	BD	38.75 ± 3.42 *
NiTa	6.26 ± 0.21	9.68 ± 0.68	5.49 ± 0.39 *	BD
CoTa	6.27 ± 0.20	11.19 ± 0.94	2.32 ± 0.17 *	BD
WNiTa	5.68 ± 0.12	5.28 ± 0.58 *	11.58 ± 2.09 *	19.33 ± 1.93 *
WCoTa	12.97 ± 0.37 *	5.99 ± 0.98 *	35.88 ± 5.11 *	76.60 ± 3.95 *
NiCoTa	5.31 ± 0.11	4.35 ± 0.92 *	BD	BD
**24-Month Groups**				
Group	Cobalt	Nickel	Tantalum	Tungsten
Control	5.78 ± 0.23	62.72 ± 2.81	BD	BD
Ta	6.24 ± 0.46	63.59 ± 4.26	BD	BD
WTa	7.42 ± 0.28	69.43 ± 3.36	8.53 ± 1.54 *	52.64 ± 5.84 *
NiTa	6.61 ± 0.26	47.78 ± 5.75 *	BD	BD
CoTa	6.71 ± 0.17	32.77 ± 2.52 *	BD	BD
WNiTa	6.19 ± 0.47	39.35 ± 3.22 *	9.16 ± 1.63 *	31.46 ± 2.86 *
WCoTa	11.19 ± 0.34 *	31.48 ± 4.62 *	38.91 ± 7.38 *	41.03 ± 4.07 *
NiCoTa	6.19 ± 0.47	19.94 ± 1.24 *	BD	BD

All mice were implanted with 4 pellets of test metal. Data represent the mean and standard error of the mean of 10 independent measurements and are expressed as ng metal per g tissue. An * indicates a result that is statistically different from the control (sham) at $p < 0.05$ using one-way ANOVA. BD, below the limit of detection. ND, not determined.

Table S18. Urine metal levels.

Group	Cobalt	Nickel	Tantalum	Tungsten
**1-Month Groups**				
Group	Cobalt	Nickel	Tantalum	Tungsten
Control	529.52 ± 92.40	7038.55 ± 1375.79	515.02 ± 114.08	711.84 ± 188.11
Ta	126.35 ± 14.38 *	2373.97 ± 357.72 *	278.43 ± 49.34	183.41 ± 27.20 *
WTa	72.74 ± 8.84 *	512.88 ± 53.70 *	361.43 ± 46.11	42,038.20 ± 6061.62 *
NiTa	84.70 ± 9.31 *	493.55 ± 262.60 *	704.16 ± 163.55	936.09 ± 178.05
CoTa	127.14 ± 21.20 *	1046.35 ± 299.18 *	661.70 ± 244.10	652.87 ± 129.09
WNiTa	140.82 ± 17.26 *	9919.40 ± 1453.34 *	326.26 ± 59.46	16,245.53 ± 3090.67 *
WCoTa	4949.43 ± 663.44 *	795.18 ± 139.84 *	307.76 ± 47.31	90,057.41 ± 13,901.72 *
NiCoTa	113.90 ± 17.84 *	885.88 ± 84.00 *	139.77 ± 23.71 *	39.91 ± 14.05 *
**3-Month Groups**				
Group	Cobalt	Nickel	Tantalum	Tungsten
Control	145.18 ± 37.30	4658.99 ± 1174.54	132.34 ± 38.81	BD
Ta	67.96 ± 17.50	1442.55 ± 477.04	50.07 ± 13.17	BD
WTa	87.08 ± 15.20	1649.12 ± 400.65	84.43 ± 12.49	37,004.38 ± 5200.32 *
NiTa	98.81 ± 23.26	1710.88 ± 653.25	74.29 ± 19.95	BD
CoTa	95.41 ± 26.54	650.95 ± 188.22 *	26.68 ± 4.63 *	BD
WNiTa	97.31 ± 27.24	3326.19 ± 836.73	77.45 ± 16.12	35,229.64 ± 6566.33 *
WCoTa	3947.50 ± 1477.85 *	2062.42 ± 853.71	438.68 ± 119.70 *	332,719.59 ± 18,890.27 *
NiCoTa	69.27 ± 21.00	1001.72 ± 245.92 *	16.48 ± 4.30 *	BD
**6-Month Groups**				
Group	Cobalt	Nickel	Tantalum	Tungsten
Control	52.63 ± 6.63	579.46 ± 50.95	BD	BD
Ta	59.29 ± 5.67	533.21 ± 74.50	11.54 ± 1.92 *	BD
WTa	81.35 ± 28.26	1116.82 ± 234.48	56.94 ± 18.77 *	25,921.94 ± 5390.63 *
NiTa	66.93 ± 14.66	1129.47 ± 190.24	19.82 ± 3.65 *	BD
CoTa	67.58 ± 13.39	962.20 ± 165.72	15.97 ± 1.63 *	BD
WNiTa	62.91 ± 16.06	945.21 ± 189.75	45.33 ± 11.70 *	21,440.46 ± 4566.34 *
WCoTa	793.01 ± 104.07 *	635.63 ± 97.50	142.05 ± 19.40 *	137,467.37± 22,137.54 *
NiCoTa	40.54 ± 6.07	1103.62 ± 242.75	21.30 ± 1.40 *	BD
**12 Month Groups**				
Group	Cobalt	Nickel	Tantalum	Tungsten
Control	111.27 ± 11.28	1051.19 ± 130.00	20.27 ± 5.07	BD
Ta	142.08 ± 19.99	978.96 ± 35.09	21.87 ± 4.29	BD
WTa	103.42 ± 8.27	1440.33 ± 265.38	61.29 ± 6.11 *	55,870.49± 7250.50 *
NiTa	69.67 ± 5.52	760.25 ± 66.16 *	22.47 ± 2.04	BD
CoTa	39.75 ± 4.34	445.70 ± 72.38 *	18.30 ± 3.16	BD
WNiTa	57.92 ± 6.02	1049.79 ± 89.74	27.27 ± 5.60	12,066.56± 2223.34 *
WCoTa	470.24 ± 77.13 *	1533.29 ± 295.06	113.49 ± 19.99 *	128,128.73± 19,993.53 *
NiCoTa	55.28 ± 7.93	348.72 ± 60.82 *	12.89 ± 4.16	BD

Table S18. *Cont.*

24-Month Groups				
Group	Cobalt	Nickel	Tantalum	Tungsten
Control	41.79 ± 5.13	393.59 ± 57.71	BD	BD
Ta	30.01 ± 3.34	320.77 ± 29.36	460.78 ± 108.68 *	BD
WTa	38.00 ± 2.46	478.85 ± 46.06	73.33 ± 3.29 *	12,089.26 ± 1618.85 *
NiTa	45.16 ± 8.65	373.10 ± 53.91	58.46 ± 4.72 *	BD
CoTa	26.97 ± 3.94	267.30 ± 34.42	16.24 ± 1.96 *	BD
WNiTa	33.78 ± 4.93	365.90 ± 23.52	59.06 ± 9.49 *	5782.47 ± 611.02 *
WCoTa	55.06 ± 4.20	308.56 ± 28.99	59.66 ± 5.56 *	7296.71 ± 723.75 *
NiCoTa	48.26 ± 8.98	392.05 ± 56.09	7.16 ± 3.23 *	BD

All mice were implanted with 4 pellets of test metal. Data represent the mean and standard error of the mean of 10 independent measurements and are expressed as ng metal per mg creatinine. An * indicates a result that is statistically different from the control (sham) at $p < 0.05$ using one-way ANOVA. BD, below the limit of detection.
